# Use of tuberculin skin test for assessment of immune recovery among previously malnourished children in Ethiopia

**DOI:** 10.1186/s13104-017-2909-x

**Published:** 2017-11-07

**Authors:** Paluku Bahwere, Philip James, Alemseged Abdissa, Yesufe Getu, Yilak Getnet, Kate Sadler, Tsinuel Girma

**Affiliations:** 1 Valid International, 35, Leopold Street, Oxford, OX4 1TW UK; 2 Research Centre in Epidemiology, Biostatistics and Clinical Research, School of Public Health, Free University of Brussels, Brussels, Belgium; 3 London School of Hygiene and Tropical Medicine, London, UK; 4 Department of Medical Laboratory Sciences and Pathology, Jimma University, Jimma, Oromia Ethiopia; 5 Save Children Federation, Addis Ababa, Ethiopia; 6 Department of Paediatrics and Child Health, Jimma University, Jimma, Oromia Ethiopia

**Keywords:** Severe acute malnutrition, Immunity, Delayed-type hypersensitivity response, Tuberculin skin test, Morbidity, Tuberculin purified protein derivative, Community-based management of acute malnutrition

## Abstract

**Objective:**

To compare levels of immunity in children recovering from severe acute malnutrition (cases) against those of community controls (controls).

**Results:**

At baseline children recovering from severe acute malnutrition had lower, mid upper arm circumference (122 mm for cases and 135 mm for controls; p < 0.001), weight-for-height Z-score (− 1.0 for cases and − 0.5 for controls; p < 0.001), weight-for-age Z-score (− 2.8 for cases and − 1.1 for controls; p < 0.001) and height/length-for-age Z-score (− 3.6 for cases and − 1.4 for controls; p < 0.001), than controls. Age and gender matched community controls. At baseline, prevalence of a positive tuberculin skin test, assessed by cutaneous delayed-type hypersensitivity reaction skin test, was very low in both cases (3/93 = 3.2%) and controls (2/94 = 2.1%) and did not significantly increase at 6 months follow up (6/86 = 7.0% in cases and 3/84 = 3.4% in controls). The incidences of common childhood morbidities, namely fever, diarrhoea and cough, were 1.7–1.8 times higher among cases than controls. In conclusion, these results show that tuberculin skin test does not enable any conclusive statements regarding the immune status of patients following treatment for severe acute malnutrition. The increased incidence of infection in cases compared to controls suggests persistence of lower resistance to infection even after anthropometric recovery is achieved.

## Introduction

Globally, 5.8 million children younger than 5 years died in 2015 and millions more are permanently disabled by the physical and mental effects of a poor dietary intake in the earliest months of life [1]. Approximately 2% of children living in low income countries suffer from severe acute malnutrition (SAM) [2]. SAM is believed to account for at least 4.4% of the global under-five death burden and 6.0% of disability-adjusted life-years lost [1]. Despite the tremendous progress of recent years, Ethiopia remains one of the countries with the highest burden of SAM [3].

The rapid scale-up of the community-based management of acute malnutrition (CMAM) approach in many high burden countries has enabled better treatment coverage and has contributed to saving lives of millions of children with SAM [4–11]. However, several studies have reported that children who recovered from SAM remain at high risk of infections and death several months after exiting treatment programmes [12–19]. One hypothesis is that the anthropometric and clinical discharge criteria that are used in treatment programmes may have weak correlation with optimal recovery of body functions including immunity.

In Ethiopia, children with SAM are discharged from CMAM as recovered when they have fulfilled the following criteria: weight gain of 20%, mid upper-arm circumference (MUAC) > 11.0 cm, resolved oedema and clinically stable for 2 consecutive weeks [20]. Here we describe a study conducted in Jimma Zone, Eastern Ethiopia, using a tuberculin purified protein derivative (PPD) skin test to verify if children who reached these discharge criteria had also recovered their immune response. This paper presents the results and discusses the utility of the test for this purpose.

## Main text

### Methods

This research was a sub-study of a larger 12 months prospective cohort study conducted from September 2013 to January 2015. The main study aimed to describe the mortality, morbidity and nutritional status profiles of children aged 6–59 months discharged as recovered from CMAM for SAM treatment. It enrolled 215 post-SAM cases (‘cases’) discharged as recovered from CMAM sites of Jimma area and 215 non-wasted community controls (‘controls’) matched from the same village with the cases by age, sex, mother’s education and roof material. The controls were apparently healthy children with no history of treatment for acute malnutrition. Cases and controls were followed up concurrently at their homes each month, assessing nutritional status (weight, height, MUAC, bilateral pitting oedema) reported morbidity of the past 2 weeks (history of fever, diarrhoea or cough) and vital status (alive or dead).

The extent of immune system recovery was assessed using the difference in incidence of infectious diseases between cases and controls and by a cutaneous delayed-type hypersensitivity reaction skin test (DTHR) at enrolment and at the 6-month follow up. The DTHR was assessed using the Tuberculin skin test (TST) [21]. TST was chosen because of its practicality for community-based surveys, its commercial availability, and its wide usage in DTHR response and immune diagnosis of tuberculosis infection [22–24]. We used Tuberculin PPD RT23 (Statens Serum Institute, Copenhagen, Denmark). The tuberculin PPD was kept refrigerated and transported to the field on ice and avoiding light exposure. This maintained the temperature of the vials between 2 and 8 °C throughout. The DTHR tests were performed by trained research nurses. 0.1 mL (2 Tuberculin Units/0.1 mL) of antigen was injected into the intradermal space on the volar surface of the forearm, using a 27-gauge needle with the bevel up and at an angle of 5–15°. Adherence to the injection technique was confirmed by the apparition of a small wheal of 6–10 mm as the antigen was injected into the dermis and by the absence of bleeding. Reading and classification of the DTHR response was based on the diameter of the induration measured 48–72 h after the antigen injection [25]. A TST was regarded as positive if the transversal diameter of skin induration was ≥ 10 mm. Children were classified as anergic if there was no induration.

Morbidity was assessed by caretaker face-to-face interviews using a 2-week recall approach and a pre-tested standardised questionnaire. Caretakers were asked if the child had experienced fever, diarrhoea, or persistent cough during 2 weeks preceding the interview. All socio-demographic, nutrition, medical and household information captured by the questionnaire followed the same methodology described in James et al. [26].

In absence of literature data allowing precise calculation of sample size, a convenience sample size of 100 cases and 100 controls was used for this study. Proportions and means were compared using Chi squared tests and unpaired Student t test, respectively. We generated incidence rate ratios to compare morbidity incidence between the cases and controls.

### Results

#### Characteristics at enrolment

Ninety-three cases and 95 controls were included in the present sub-study. The two groups did not differ for most of the parameters checked except for orphan status, age of carers, number of children below 5 years of age in the household, proportion of food secure households and nutrition status (Table 1). At enrolment the nutritional status of cases, indicated by MUAC, weight-for-height Z-score, weight-for-age Z-score and height/length-for age Z-score, were lower than that of the controls (Table 1).

**Table 1:** Socio-demographic, medical, nutrition and household characteristics of participating children

Characteristics	Post-SAM cases (n = 93)		Community controls (n = 95)		p value
	n (%)	Median (IQR)	n (%)	Median (IQR)	
Socio-demographic characteristics					
Female	39 (41.9)		41 (43.2)		0.865
Age (months)		15 (6; 48)		14 (7; 48)	0.309
Medical history					
Ever immunised	77 (82.8)		80 (84.2)		0.794
Presence of BCG scar	54 (58.1)		65 (68.4)		0.326
Recent vitamin A supplementation	86 (92.5)		87 (91.6)		0.821
Recent deworming	52 (55.9)		44 (46.3)		0.188
Utilises insecticide treated bed net	47 (50.5)		53 (55.8)		0.767
Nutrition status					
Mid upper arm circumference (mm)		122 (118; 131)		135 (130; 143)	< 0.001
Weight-for-age Z-score^a^		− 2.8 (− 3.6; − 2.2)		− 1.1 (− 1.8; − 0.5)	< 0.001
Height-for-age Z-score^a^		− 3.6 (− 4.6; − 2.6)		− 1.4 (− 2.4; -0.7)	< 0.001
Weight-for-height Z-score^a^		− 1.0 (− 1.9; − 0.4)		− 0.5 (− 1.1; 0.2)	< 0.001
% severe stunting^a^	60 (65.2)		17 (18.1)		< 0.001
Caregivers information					
Both parents alive	79 (85.9)		92 (96.8)		0.004
Mother as principal caregiver	89 (95.7)		93 (97.9)		0.442
Caregiver ever attended school	17 (18.3)		22 (23.2)		0.410
Principal caregiver age (years)		26 (13; 60)		25 (18; 55)	0.034
Caregiver MUAC (mm)		222 (182; 297)		229 (122; 267)	
Infant and child feeding index^b^					0.074
Lowest category	2 (2.1)		4 (4.2)		
Middle category	42 (45.2)		28 (29.5)		
Highest category	49 (52.7)		63 (66.3)		
Household characteristics					
Male headed household	88 (95.6)		92 (96.8)		0.668
Household head attended school	30 (32.3)		34 (36.2)		0.573
Household size		6 (2–22)		5 (2–12)	0.081
Number children below 5 years		2 (1–2)		1 (1–2)	0.020
Food secure	50 (56.2)		77 (82.8)		0.001
WASH practices category					0.789
Good WASH index^c^	55 (59.1)		58 (61.0)		
Poor WASH index	38 (40.9)		37 (39.0)		
Wealth quartiles distribution^d^	n = 88		n = 89		0.806
First	25 (28.4)		21 (23.6)		
Second	23 (26.1)		27 (30.3)		
Third	19 (21.6)		22 (24.7)		
Fourth	21 (23.9)		19 (21.3)		

*SAM* severe acute malnutrition, *MUAC* mid upper arm circumference, *IQR* interquartile range, *WASH* water, sanitation and hygiene

^a^Using 2006 world Health organisation multicentre child growth references

^b^Based on 24 h recall of breastfeeding, dietary diversity and meal frequency

^c^Summarised as recommended by WHO and UNICEF methodology [62]

^d^Generated using principal component analysis as described in [61]

#### Results of cell mediated immunity assessment

**Fig. 1:**
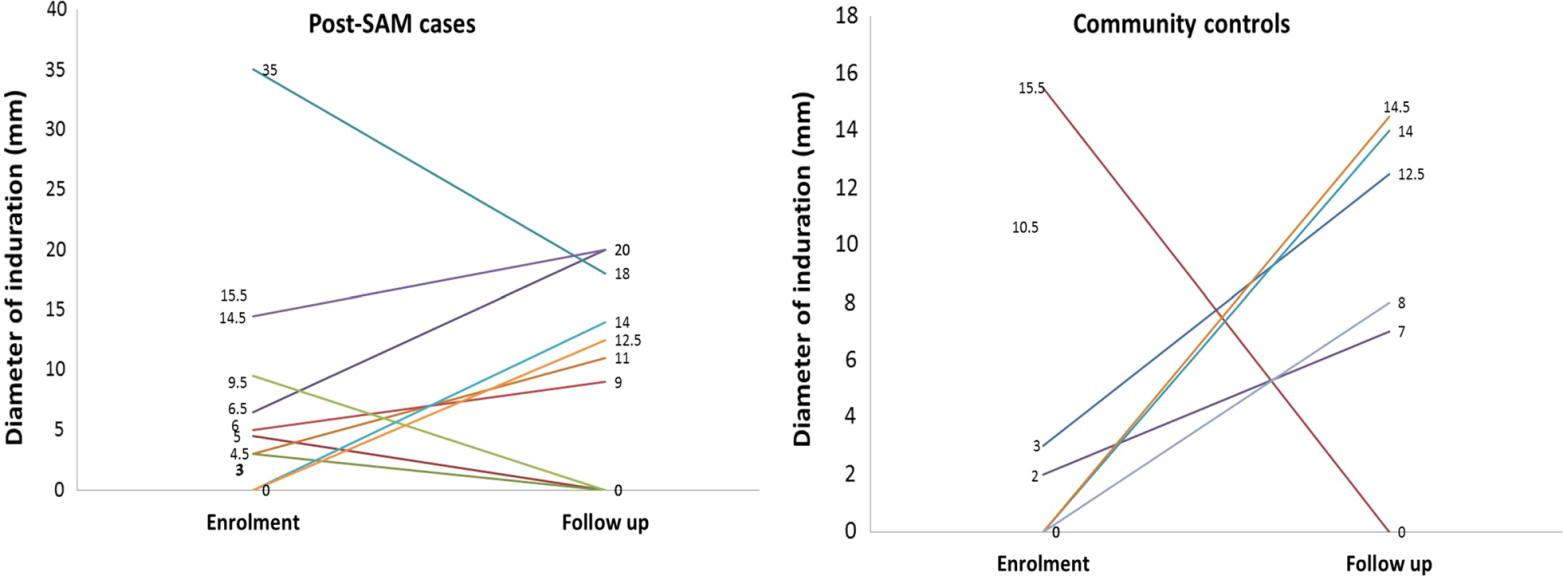
Diameter of induration at enrolment and follow up for the children exhibiting an induration during at least one assessment time-point. *Post-SAM* post-severe acute malnutrition treatment (cases)

In both cases and controls, less than 5% of children had a positive tuberculin skin test (Table 2). The rate of positivity did not increase between baseline and 6 months after enrolment (Table 2). Four cases anergic at enrolment had a positive TST test at 6-months while one who had an induration of 9.5 mm at enrolment was anergic at 6 months follow up. For the majority of reactive children, the diameter of induration was larger at 6-month follow up than at enrolment (Fig. 1).

**Table 2:** Results of delayed-type hypersensitivity reaction on tuberculin antigen

Skin test categories	Baseline	Follow-up (6 months)	p value
	n (%)	n (%)	
Proportion of anergic			
Post-SAM (n = 93/86)	83 (89.2)	79 (92.0)	0.551
Community control (n = 94/89)	90 (95.7)	84 (94.4)	0.742
p value	0.091	0.604	
Proportion with any induration			
Post-SAM (n = 93/86)	10 (10.8)	7 (8.0)	0.551
Community control (n = 94/89)	4 (4.3)	5 (5.6)	0.742
p value	0.091	0.604	
Positive test (induration ≥ 10 mm)			
Post-SAM (n = 93/86)	3 (3.2)	6 (7.0)	0.316
Community control (n = 94/89)	2 (2.1)	3 (3.4)	0.676
p value	0.682	0.324	

Skin test categories	Baseline	Follow-up (6 months)	p value
	Mean ± SD	Mean ± SD	
Average diameter of induration			
Post-SAM (n = 10/7)	10.2 ± 9.8	14.9 ± 4.4	0.256
Community control (n = 4/5)	7.7 ± 6.4	11.2 ± 3.5	0.327
p value	0.648	0.150	
Average induration for positive test			
Post-SAM (n = 3/6)	21.7 ± 1.0	15.9 ± 3.9	0.044
Community control (n = 2/3)	13.0 ± 3.5	13. 7 ± 1.0	0.748
p value	0.398	0.376	

Proportions are compared using Pearson Chi square or Fisher exact test as appropriate, means are compared using unpaired Student t test. *SAM* severe acute malnutrition, delayed-type hypersensitivity skin reaction considered positive if induration diameter ≥ 10 mm

#### Results on incidence of reported common morbidities

The incidences of fever [12.45 per 100-child-months in cases and 7.25 per 100-child-months in controls; incidence rate ratio (IRR): 1.85 (1.33–2.20); p < 0.001], diarrhoea [11.90 per 100-child-months in cases and 6.70 per 100-child-months in controls; IRR: 1.77 (1.37–2.29); p < 0.001], and cough [12.42 per 100-child-months in cases and 6.94 per 100-child-months in controls; IRR: 1.79 (1.37–2.29); p < 0.001] were 1.7–1.8 times higher among cases than controls.

### Discussion

Undernutrition in early life is associated with a number of adverse health consequences, including impaired growth and neurocognition, long-term body composition abnormalities and persistent immune system dysfunction [27–30]. Little is known about how the CMAM approach can be improved so that long-term benefits are added to the well-documented short-term benefits already being achieved. Thus, it is important to start to focus on improving our knowledge on the impact of current treatment protocols on the quality of immune system normalisation as it is clear that cell mediated immunity and resistance to infection are not being adequately recovered.

This short report presents our attempts to characterize the quality of immune system repair at the time of being discharged as recovered from CMAM programmes. Using the TST we were not able to confirm whether cell-mediated immunity was adequately or inadequately repaired in post-SAM cases as the TST appeared to be inappropriate for assessing immune system functioning in our study setting. However, the difference in incidence of symptoms of common infectious diseases suggests cases did have lowered immunity than controls, since impairment in cellular mediated immunity is independently associated with increased incidence of these diseases [31–33].

Despite the still limited understanding of the interplay between the immune function and SAM, existing evidence points toward impairment of cell-mediated immunity during SAM episode including DTHR [34–39]. Also, it has been suggested that DTHR return to normal after successful treatment of SAM [40]. Thus, we understood that checking DTHR using TST was logical to assess the immune system recovery in children who were treated under the current CMAM programme as virtually all children are Bacille Calmette–Guérin (BCG) vaccinated in the region and BCG vaccinated children with low risk of tuberculosis infection can have a positivity of up to 80%, as observed in Turkey [41]. Unfortunately, contrary to our hypothesis of high prevalence of positive DTHR in matched controls, almost all exhibited anergy making it difficult to link the impaired DTHR in cases solely to the insufficiency of nutrition recovery.

Our results differ from that of an early study conducted in Colombia that showed a rapid increase in TST positivity from 0% for kwashiorkor children and 5.5% for marasmic children at admission to 50% for kwashiorkor children and 90.9% for marasmic children after 6 weeks of therapeutic feeding [37]. They also differ from that of Forse et al. who showed in 257 adults on pre-surgical total parenteral nutrition that reduced cell mass was associated with impaired DTHR, while nutrition recovery and accompanying increased body mass cells improved the likeliness of positive DTHR [42]. However, our results are in accord with some studies conducted in African children that showed negative DTHR even in presence of both BCG scar and confirmed active tuberculosis pulmonary infections [23, 24, 43, 44]. They are also consistent with results of a study among Ethiopian adults that reported a lower prevalence of tuberculosis infection when using TST than when using T-cell based interferon-g release assays [45]. Thus, even if TST may be used in certain contexts to assess immune system competence, our study suggests it cannot be universally recommended for children (whether these are children with SAM or those who are apparently well-nourished) living in countries with similar characteristics as Ethiopia. We tentatively suggest that in rural Ethiopia the high frequency of environmental enteric dysfunction and high incidence of viral, bacterial and parasitic infection may adversely affect the DTHR test, including in non-malnourished children, by maintaining children in a state of chronic inflammation [22, 34, 46–48]. Also, despite not being wasted, the control group may have undiagnosed micronutrient deficiencies that can affect the DTHR [49]. These factors may also explain why many of the children did not have a BCG scar despite being BCG vaccinated. Nonetheless, TST negative results are also common among BCG vaccinated children from United Kingdom, who are unlikely to have micronutrients deficiencies and who are most likely to be immune-competent, suggesting that factors unrelated to immunity contribute to the presence of the negative TST [50].

Despite the null TST results the fact that cases had a higher reported incidence of common diseases than controls suggests that at time of reaching the anthropometric discharge criteria currently recommended by the Ethiopian national guidelines for management of SAM, the immunity has not completely recovered. This is in accord with several previous studies that showed that immune recovery measured by thymus size, serum immunoglobulin level or CD4 count is delayed comparatively to anthropometric recovery [51–54]. To date, all these results have not yet been considered for defining practical criteria for judging recovery that combine body mass catch up and immune recovery [20, 55].

In conclusion, this study did not permit a conclusion on immune recovery following CMAM treatment for SAM, whether at discharge or at 6 months follow up but suggests persistence of lower resistance to infection after the anthropometric criteria of nutrition recovery are reached. Research is needed to identify a clinical proxy of immunity recovery that can be used in community-based programmes in low income countries.

## Limitations

Having a laboratory blood test confirming immunity disturbance could have strengthened our conclusion. Rytter et al. have proposed laboratory tests to use in the assessment of immune system recovery, but most of the proposed tests are difficult to conduct in the low income settings where most SAM children are found [34]. Future research should include the quantification of T cell receptor excision circles using Dried Blood Spot specimens to measure the quantity of circular deoxyribonucleic acid molecules formed during rearrangement of the T cell receptor genes during lymphocyte development [56–58]. The test is increasingly used in screening newborns for primary immunodeficiency and for assessing immune response to the antiretroviral treatment of HIV infected children [59, 60]. The simplicity of the sampling allows prospective sampling at several points during management and follow-up after discharge and may help validate the choice of clinical and anthropometric parameters best used as a proxy of immune system recovery.

## Abbreviations

BCG: Bacille Calmette–Guérin
CMAM: community-based management of acute malnutrition
DTHR: delayed-type hypersensitivity reaction
ENGINE: empowering new generations to improve nutrition and economic opportunities
MUAC: mid upper-arm circumference
PPD: tuberculin purified protein derivative
SAM: severe acute malnutrition
TST: tuberculin skin test
